# Macrophage-Derived Angiopoietin-Like Protein 2 Exacerbates Brain Damage by Accelerating Acute Inflammation after Ischemia-Reperfusion

**DOI:** 10.1371/journal.pone.0166285

**Published:** 2016-11-18

**Authors:** Toshihiro Amadatsu, Jun Morinaga, Takayuki Kawano, Kazutoyo Terada, Tsuyoshi Kadomatsu, Keishi Miyata, Motoyoshi Endo, Daiki Kasamo, Jun-ichi Kuratsu, Yuichi Oike

**Affiliations:** 1 Department of Molecular Genetics, Graduate school of Medical Sciences, Kumamoto University, 1-1-1 Honjo, Chuo-ku, Kumamoto, 860–8556, Japan; 2 Department of Neurosurgery, Graduate School of Medical Sciences, Kumamoto University, 1-1-1 Honjo, Chuo-ku, Kumamoto, 860–8556, Japan; Texas Tech University, UNITED STATES

## Abstract

Ischemic stroke is a leading cause of death and disability worldwide. Several reports suggest that acute inflammation after ischemia-reperfusion exacerbates brain damage; however, molecular mechanisms underlying this effect remain unclear. Here, we report that MAC-3-positive immune cells, including infiltrating bone marrow-derived macrophages and activated microglia, express abundant angiopoietin-like protein (ANGPTL) 2 in ischemic mouse brain in a transient middle cerebral artery occlusion (MCAO) model. Both neurological deficits and infarct volume decreased in transient MCAO model mice established in *Angptl2* knockout (KO) relative to wild-type mice. Acute brain inflammation after ischemia-reperfusion, as estimated by expression levels of pro-inflammatory cytokines such as interleukin (IL)-1β and tumor necrosis factor alpha (TNF)-α, was significantly suppressed in *Angptl2* KO compared to control mice. Moreover, analysis employing bone marrow chimeric models using *Angptl2* KO and wild-type mice revealed that infiltrated bone marrow-derived macrophages secreting ANGPTL2 significantly contribute to acute brain injury seen after ischemia-reperfusion. These studies demonstrate that infiltrating bone marrow-derived macrophages promote inflammation and injury in affected brain areas after ischemia-reperfusion, likely via ANGPTL2 secretion in the acute phase of ischemic stroke.

## Introduction

The number of people who experience ischemic stroke or stroke-related permanent brain damage is increasing worldwide.[1] Because ischemia rapidly and irreversibly impairs central nervous system (CNS) function, early intervention to reperfuse the ischemic region by intravenous injection of tissue plasminogen activator or thrombectomy has been globally approved in clinical medicine.[1]

Perturbation of blood flow by transient occlusion and reperfusion damages brain structures and component cells, including neurons and glial cells, through hypoxia, production of reactive oxygen species and promotion of inflammation.[2] Interestingly, recent evidence suggests that acute inflammation driven by activated immune cells significantly exacerbates brain injury in ischemic brain.[3, 4] Immediately after occlusion of a vessel, the brain’s initial innate response is triggered primarily by microglia cells, which reportedly function as resident macrophages of the CNS.[5] However, once the vascular network known as the blood brain barrier (BBB) is disrupted by ischemia, circulating or perivascular macrophages infiltrate the parenchyma of brain tissue.[3] Activated infiltrated macrophages cause neuronal injury by amplifying inflammation via secretion of pro-inflammatory cytokines such as interleukin (IL)-1β[6, 7] or tumor necrosis factor (TNF)-α[8]. Sustained inflammation in ischemic brain caused by pro-inflammatory cytokines further injures neurons.[9–11] Therefore, in combination with reperfusion therapy, strategies to suppress inflammation caused by these factors could represent an attractive therapy to attenuate neuronal damage.

Angiopoietin-like proteins (ANGPTLs), which possess an N-terminal coiled coil domain used for oligomerization and C-terminal fibrinogen-like domain, are a family of secreted proteins structurally similar to Angiopoietin but which do not bind the Angiopoietin receptor tyrosine kinase with Ig-like and EGF-like domains (Tie) 2 or the related receptor Tie 1.[12] We have reported that among ANGPTLs, ANGPTL2 functions in physiological tissue remodeling and plays crucial roles in pathological conditions associated with chronic non-infectious inflammation, including obesity-related adipose tissue inflammation, atherosclerotic vascular disease, carcinogenesis and cancer metastasis.[13–17] However, little is known about ANGPTL2 function in acute inflammation.[8]

Here, we report a molecular mechanism whereby bone marrow-derived macrophages promote acute brain damage after ischemic stroke. Our study suggests that ANGPTL2 secretion by bone marrow-derived infiltrating macrophages enhances inflammation in acute ischemic brain by promoting abundant expression of pro-inflammatory cytokines including IL-1β or TNF-α and provides insight into molecular mechanisms underlying neuronal injury in acute ischemic brain.

## Materials and Methods

### Animals

All procedures of the animal study were approved by the Kumamoto University Ethics Review Committee for Animal Experimentation. Eight to ten weeks old male animals were bred in a mouse house with automatically controlled lighting (12 hours on, 12 hours off), and a stable temperature of 23°C was maintained throughout. Mice were fed a normal diet (CE-2, CLEA Japan, Tokyo, Japan). Wild-type (WT) and *Angptl2* knockout (KO) mice on a C57BL/6N background were used in all experiments as described.[16]

### Mouse focal brain ischemia model

Male mice, aged 8–10 weeks and weighing 18-25g, were used for the transient middle cerebral artery occlusion (MCAO) model, which was performed as per *Kawano* et al.[18] Briefly, mice were anesthetized with 1.5% isoflurane and rectal temperatures were maintained at 37.0°C using a heating pad (Muromachi Kikai, Tokyo, Japan). Cerebral blood flow (CBF) at the ipsilateral parietal bone (~1–2 mm posterior to bregma) was measured before and during ischemia by laser Doppler flowmetry (Muromachi Kikai). Resting CBF levels were assessed to provide a baseline for an individual animal, and the percent CBF reduction relative to that resting value was calculated during brain ischemia. After ligation of the right common carotid artery (CCA), the right middle cerebral artery (MCA) was occluded with a 6–0 nylon monofilament with a silicone rubber tip (11 mm-long, Doccol Corporation, Sharon, MA)[19]. During right MCA occlusion, a greater than 80% reduction in CBF was confirmed by laser Doppler flowmetry. Thirty-minutes after ischemia, the filament was withdrawn to allow reperfusion of the right MCA territory. Mice were sacrificed by deep anesthesia, 24 and 72 hours later. Exclusion criteria for animals in the transient MCAO model and the number of excluded mice in *in vivo* experiments are indicated in the S1A Appendix.

Sample size in animal experiments was calculated from pilot study data. In calculating mouse number, α error was set to 5% and β error to 20%. In experiments with WT or *Angptl2* KO mice, one researcher undertook genotyping and/or prepared the bone marrow chimeric model and another, blinded to genotypes, performed the transient MCAO procedure, neurological scoring, animal sacrifice, histological analysis and real-time PCR. Statistical analysis was performed after genotypes were revealed.

### Generation of rabbit monoclonal antibody (RabMAb) to mouse ANGPTL2 and antigen preparation

Recombinant mouse ANGPTL2 C-terminally tagged with hexahistidine (constructed in the pQE vector (Qiagen, Hilden, Germany)) was expressed in *E*. *coli* RosettaTMpLacl cells (Merck, Kenilworth, NJ, USA). Induced protein in inclusion bodies was washed thoroughly and solubilized in solution containing 6M guanidine hydrochloride and then reduced in 10 mM dithiothreitol and modified by 3-trimethylammoniopropyl methanethiosulphonate bromide (TAPS; Wako, Osaka, Japan), according to a published procedure {Terzyan, 1999 #107}. TAPS-modified proteins were desalted, and recombinant protein was purified by metal chelating chromatography with a Talon affinity column (Takara Bio). A portion of the purified protein was refolded by dilution into a solution of a mixed disulfide (2 mM cysteine and 0.5 mM cystine) and 2M sodium lactate, pH 8.0, at 4°C for 14 h under a nitrogen atmosphere. Refolded protein was desalted by reverse-phase chromatography with a Source 30 matrix (GE Healthcare, Little Chalfont, UK). The sample, eluted in acetonitrile-0.04% trifluoroacetic acid buffer, was freeze-dried and dissolved in 0.1% acetic acid. Samples with an endotoxin level <0.3 EU/mg were stored at -80°C until immunization.

The ANGPTL2 RabMAb was generated by a custom service (Abcam, Cambridge, MA, USA). A single hybridoma clone (2E3) was selected for immunostaining.

### Immunohistochemistry

Mouse brain tissue samples were fixed in 4% paraformaldehyde for 24 hours and embedded in paraffin. Blocks were cut into 5-μm-thick sections, air-dried, and deparaffinized. Sections were autoclaved for antigen retrieval. For single immunohistochemistry for ANGPTL2, sections were incubated with 200-fold diluted rabbit monoclonal anti-mouse ANGPTL2 antibody described above. Sections were incubated overnight at 4°C and washed with PBST. A modified protocol for tyramide signal amplification (TSA-Biotin Systems; PerkinElmer, 940 Winter Street Waltham, MA) was subsequently followed. Sections were incubated with 200-fold diluted anti-rabbit IgG conjugated with biotin as second antibody at room temperature for 30min, washed with PBST, incubated with 100-fold diluted streptavidin-horseradish peroxidase conjugate at room temperature for 30min, and washed again with PBST. Specimens were incubated with biotin tyramide for 10min and washed with PBST. Sections were incubated with 100-fold diluted streptavidin-horseradish peroxidase, washed again with PBST, and then peroxidase activity was visualized by incubation with a 3, 3-diaminobenzidine solution. Specimens were then analyzed by light microscopy.

For MAC-3 immunostaining, sections were autoclaved and incubated with anti-MAC-3 antibody (1:100) (BD Biosciences, Erembodegem, Belgium) overnight at 4°C and then washed with PBST buffer. Sections were incubated with anti-rat IgG conjugated with peroxidase (Simple Stain Mouse MAX-PO, Nichirei, Japan) as second antibody at room temperature for 60min. Peroxidase activity was visualized by incubation with a 3, 3-diaminobenzidine solution (DAKO). Specimens were analyzed by light microscopy.

### Brain microvascular density evaluation

For the angiogenesis assay, 8-week-old mice (5 wild-type and 4 *Angptl2* KO) were deeply anesthetized and perfused intracardinally with 20ml PBS. Brains were dissected out and frozen in Tissue-Tek O.C.T. compound (Sakura Finetek, Tokyo, Japan) in liquid nitrogen. Blocks were cut into 6 μm-thick sections, which were fixed and incubated with cold acetone for 20 min. After washing in PBS, sections were blocked in 5% goat serum for 20 minutes at room temperature and incubated with 1:100 anti-CD31 antibody (BD Pharmingen) overnight at 4°C. Sections were then incubated with Histofine Simple Stain MAX PO (Rat) (Nichirei, Tokyo, Japan) for 60 min at room temperature and signals visualized with diaminobenzidine and hydrogen peroxide. Sections were counterstained with hematoxylin.

Brain microvascular density was evaluated in wild type (n = 5) and *Angptl2* KO (n = 4) mice by light microscopy at x400 magnification (PMID: 15265595, 15509618). Ten pictures were randomly obtained from the right caudate putamen, and CD31-positive cells were counted in samples.

RNA was extracted using TRI Reagent (Molecular Research Center, Inc.) and converted into cDNA using PrimeScript RT master mix (TaKaRa Bio, Osaka, Japan) as per the manufacturer’s instructions. SYBR Green for *Pecam-1* and *Tbp* were purchased from TaKaRa Bio. PCR products were analyzed using a Thermal Cycler Dice Real Time system (Takara Bio), and transcript abundance was normalized to that of *Tbp* mRNA. Oligonucleotides used for PCR are listed in S2 Appendix.

### Neurological assessment

Twenty four hours after transient MCAO, neurological function was evaluated using an 18-point neurological severity scale (S3 Appendix, Garcia scale[20]) before mice were sacrificed.

### Measurement of infarct volume

One-millimeter-thick serial coronal brain slices were stained with 2% triphenyl tetrazolium chloride (TTC) to measure infarct volume. The infarct region was identified as an unstained area using by this procedure [21]. To calculate volume of whole brain ischemic damage, all areas (mm^2^) of brain ischemia per slice (as evaluated by ImageJ software (National Institutes of Health, Bethesda, MD) were multiplied by 1mm slice thickness to calculate volumes, which were summed.

### Droplet digital PCR

RNAs from mouse brain or cultured cells were extracted using TRI Reagent (Molecular Research Center, Cincinnati, OH) and converted into cDNA using PrimeScript RT master mix (TaKaRa, Shiga, Japan) according to the manufacturer’s instructions. Droplet digital PCR (ddPCR) for *Il1b*, *Tnfa*, *Il10* and *Tbp* transcripts was carried out using a QX200 ddPCR system (Bio-Rad Laboratories, Hercules, CA) according to the manufacturer’s instruction. Data were analyzed with QuantaSoft analysis software (Bio-Rad), and target molecules were quantified as copies/μl of PCR reaction. *Il1b*, *Tnfa*, *Il10* levels were normalized to *Tbp* levels.

### Quantification of MAC-3-positive cells

MAC-3-positive cells in brain were evaluated in WT (n = 14) and *Angptl2* KO (n = 6) mice by light microscopy at x400 magnification. Ten pictures were randomly obtained from cerebral hemispheres, and MAC-3-positive cells were counted in samples.

### Bone marrow-chimeric mice

Recipient mice were administered lethal doses of total body radiation with 9-Gy exposure and then rescued by tail vein injection of donor bone marrow cells. To alleviate pain, recipient mice were delivered isoflurane via inhalation during intravenous transfer of bone marrow cells from donor mice. After irradiation, mice were then monitored mice daily to check for overt signs of suffering. Body weight of bone marrow chimeric mice was assessed on postoperative days 0, 3, 7, 14, 21 and 28.

If mice appeared unwell, we increased amount of bedding in cages and placed water/food in closer proximity to animals. To reach a humane endpoint, we generally observed criteria established by “Recognition and Alleviation of Pain in Laboratory Animals” [22]. Briefly, we monitored rapid weight loss (≥20% body weight), extended period of weight loss progressing to emaciation, surgical complications, unresponsiveness to medication, and any combination of the following: poor physical appearance, reduced mobility/unconsciousness, severe depression or abnormal response to external stimuli, severe respiratory distress, serious injury, bleeding from any orifice, and skin ulcers that did not heal.

Before the experimental endpoint, 12 animals died. We did not observe trauma or bleeding and believe that most died of bacterial infection [23]

### Cell culture

The mouse macrophage line RAW264.7 was cultured in RPMI-1640 (Wako, Osaka, Japan) containing 10% FCS in 5% CO/95% air at 37°C. All experiments were performed at 70% confluence. RAW264.7 cells were first maintained 12 hours in RPMI 1640 containing 1% FCS. Cells were then treated with 5μg/mL recombinant ANGPTL2 protein and harvested at 0, 3, 6, 12 and 24 hours later.

### Real-time PCR of RAW264.7 cells

RNA from RAW264.7 cells was extracted using an RNeasy mini kit (Qiagen, Venlo, Netherlands), and 100 ng of total RNA was reverse-transcribed. PCR products were analyzed with a Thermal Cycler Dice Real Time system (Takara Bio), and relative transcript levels were normalized to *Actb*. Oligonucleotides used for PCR are listed in S2 Appendix.

### Statistical analysis

We performed one-way analysis of variance (ANOVA) followed by post hoc multiple-comparison tests (Dunnett’s correction) to analyze differences among three or more groups of mice. A Mann Whitney U test was performed to determine statistical significance between two groups in comparing neurological deficit scores, and Student's *t*-tests were used for comparisons between other groups. *P*<0.05 was considered a significant difference. Statistical analyses were performed using GraphPad Prism version 6.0 for Windows (GraphPad Software, San Diego, CA)

## Results

### ANGPTL2 levels increase in ischemic brain

**Fig 1:**
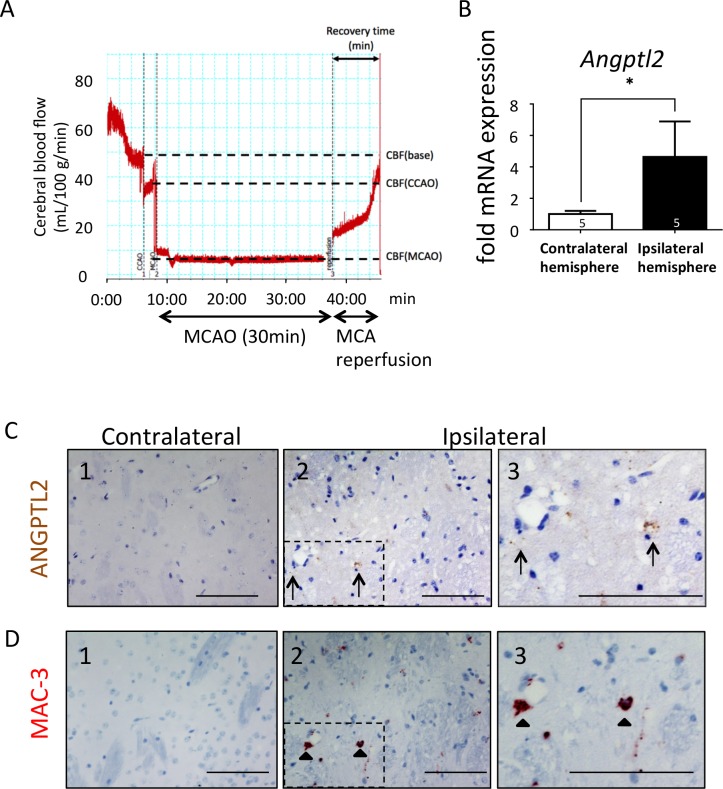
The number of infiltrated ANGPTL2-positive macrophages increases in ischemic brain. (**A**) Representative image of cerebral blood flow measured by laser Doppler flowmeter in the vicinity of the middle cerebral artery during transient middle cerebral artery occlusion (MCAO) surgery. MCA, middle cerebral artery. A laser Doppler flowmetry probe was used to trace cerebral blood flow (CBF). After ligation of the right common carotid artery (CCAO), we observed a 40% drop in CBF. MCA occlusion promoted a further decrease to less than 80–90% of baseline CBF. After 30 min MCAO, the suture was removed and the right common carotid artery was permanently ligated to prevent bleeding, resulting in a slow return of CBF to approximately 60% of baseline levels. CBF tracing was comparable in WT and *Angptl2* KO mice during MCAO and subsequent reperfusion. (**B**) *Angptl2* mRNA levels in the ischemic hemisphere 24 hours after transient MCAO (n = 5). Values indicate *Angptl2* expression relative to *Tbp* mRNA. Results are expressed as means ± s.e.m. ^*^P<0.05. (**C**) ANGPTL2 immunostaining in mouse brain. (Panel 1) Representative image showing ANGPTL2 expression in the contralateral cerebral hemisphere. (Panels 2, 3) Representative images showing ANGPTL2 expression in the ipsilateral hemisphere. Panel 3 is higher magnification image of the square in panel 2. Arrows indicate ANGPTL2-positive cells. (**D**) MAC-3 immunostaining in mouse brain. (Panel 1) Representative image showing MAC-3 expression in the serial section shown in panel 1 of (**C**). (Panels 2, 3) Representative images of MAC-3 in serial sections shown in panels 2 and 3 of (**C**). Panel 3 is a higher magnification image of the square shown in panel 2. Arrowheads indicate MAC-3-positive cells. Scale bars: 100 μm.

To evaluate whether ANGPTL2 expression changes in conditions of acute brain ischemia, we evaluated *Angptl2* mRNA levels in the affected cerebral hemisphere of forebrain compared to the contralateral hemisphere in a mouse transient middle cerebral artery occlusion (MCAO) model. In that model, cerebral blood flow in the region of the middle cerebral artery (MCA) was markedly decreased in all mice (Fig 1A). Twenty-four hours later, transient MCAO had significantly increased *Angptl2* mRNA expression in the ipsilateral compared to contralateral hemisphere (Fig 1B).

To identify cells expressing ANGPTL2 in the acute ischemic brain, we performed ANGPTL2 immunohistochemistry in both the affected and contralateral cerebral hemispheres of the forebrain in transient MCAO-treated mice. Analysis revealed abundant ANGPTL2 expression in choroid plexus and ependyma of the contralateral cerebrum of mice before MCAO treatment (S5 Appendix). Transient MCAO did not significantly alter ANGPTL2 expression in contralateral hemisphere (data not shown). On the other hand, transient MCAO significantly increased the number of ANGPTL2-positive cells in the affected cerebral hemisphere (Fig 1C, panels 2 and 3), while a significant number of ANGPTL2-positive cells was not observed on the healthy side (Fig 1C, panel 1). Moreover, immunohistochemistry for the macrophage and microglia marker MAC-3 (CD107b) [24] in serial sections of the affected hemisphere revealed ANGPTL2 expression is in MAC-3-positive cells (Fig 1D, panels 1, 2, and 3). These data suggest that the number of ANGPTL2-positive macrophages either infiltrated from the circulation or present as resident activated microglia increases in ischemic brain, accounting for increased *Angptl2* mRNA expression seen in the ipsilateral relative to the contralateral hemisphere.

### *Angptl2*-deficient mice show decreased ischemic brain injury than do wild-type mice in a transient MCAO model

To determine ANGPTL2 function in ischemic stroke, we compared wild-type (WT) and systemic *Angptl2* knockout (KO) mice in terms of degree of cerebral injury in the transient MCAO model. To do so, we first carefully compared gross structure of brain vessels in WT and KO mice prior to MCAO and observed no significant difference between genotypes (S6A Appendix). Furthermore, we performed immunostaining for CD31, a vascular endothelial cell marker,[25] in cerebral hemispheres prior to MCAO and observed no significant difference in baseline microvascular density in WT and KO mice (S6B Appendix and S6C Appendix). Levels of *Pecam1* transcripts, which encode CD31, were also comparable in WT mice and *Angptl2* KO mice in both the cortex and caudate putamen, which will constitute the penumbra and ischemic core after MCAO, respectively (S6D Appendix).

**Fig 2:**
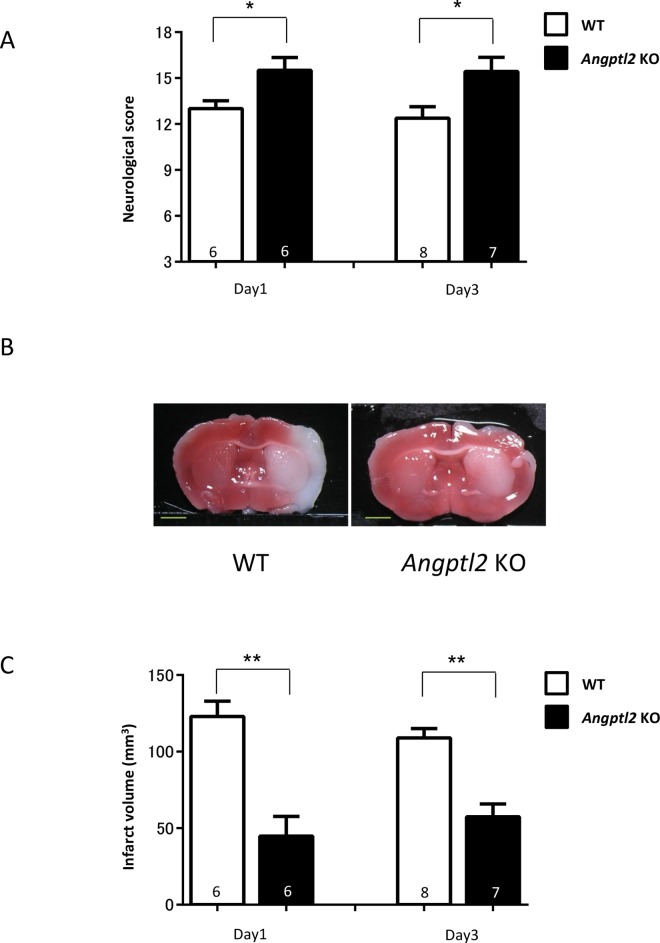
Ischemic injury is attenuated in the brain of *Angptl2*-deficient mice in a mouse transient MCAO model. (**A**) Neurological scores assessed 24 and 72 hours after transient MCAO (day 1: WT = 6 and KO = 6; day 3: WT = 8 and KO = 7) (Garcia scale, S3 Appendix). Results are expressed as means ± s.e.m. (**B**) Representative triphenyl tetrazolium chloride (TTC)-stained coronal sections from ischemic brain 24 hours after transient MCAO. Scale bars: 2mm. (**C**) Infarct volume as measured by TTC-staining of serial coronal sections 24 and 72 hours after transient MCAO (day 1: WT = 6 and KO = 6; day 3: WT = 8 and KO = 7). Results are expressed as means ± s.e.m. ^*^P<0.05, ^**^P<0.001.

We also observed no significant differences in recovery slopes following reperfusion (S7A Appendix), recovery times during transient MCAO surgery (S7B Appendix), cerebral blood flow (CBF) reduction rate after MCA occlusion (S7C Appendix), and mortality after surgery between WT and *Angptl2* KO mice in the transient MCAO model. At both 24 and 72 hours after transient MCAO, *Angptl2* KO mice showed significantly improved neurological function as evaluated using an 18-point-scale neurological score based on motor, sensory abilities and activity (18 = no observable deficit, 3 = most severe disability, see S3 Appendix) relative to WT mice (Fig 2A). ANGPTL2 levels significantly increased in ischemic brain 24 hours after MCAO (Fig 1B), likely contributing to neuronal injury seen in the acute phase of ischemic stroke. Triphenyltetrazolium chloride (TTC) staining revealed that infarct volume of MCAO treated brain (0 mm^3^ in all pre-transient MCAO mice) was significantly smaller in *Angptl*2 KO relative to WT mice, both at 24 and 72 hours after transient MCAO (Fig 2B and 2C).

### Acute brain inflammation after ischemic stroke decreases in *Angptl2*-deficient mice

**Fig 3:**
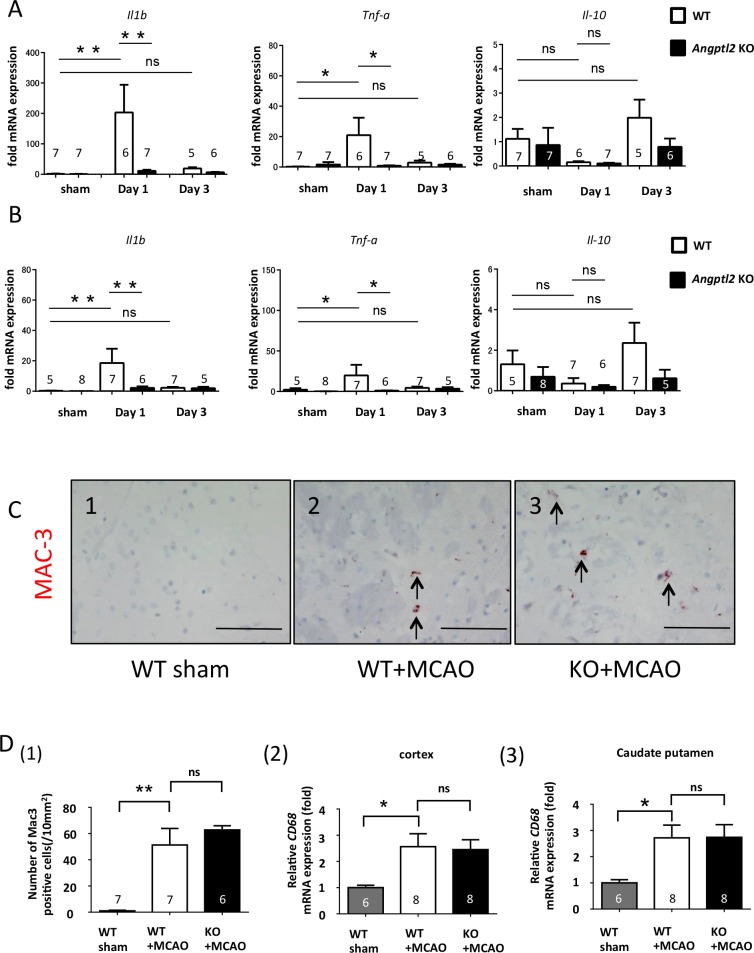
Acute brain inflammation after ischemic stroke is attenuated in *Angptl2*-deficient mice. (**A** and **B**) Relative expression of *Il1b*, *Tnfa*, and *Il10* mRNAs in cortex (sham: WT = 7 and KO = 7; day 1: WT = 6 and KO = 7; day 3: WT = 5 and KO = 6) (**A**) and ipsilateral caudate putamen (sham: WT = 5 and KO = 8; day 1: WT = 7 and KO = 6; day 3: WT = 7 and KO = 5) (**B**). mRNA levels were normalized to those of T*bp*. Values are expressed as fold-increases relative to values in WT, which were set at “1” in each case. (**C**) Representative images showing MAC-3 immunostaining in the ipsilateral cerebral hemisphere 24 hours after sham or transient MCAO treatment. (Panel 1) Sham-treated wild-type (WT) mice; (Panel 2) transient MCAO-treated wild-type mice; (Panel 3) transient MCAO-treated *Angptl2* KO mice. Scale bar: 100μm. (**D**) (1) Number of MAC-3-positive cells in the ipsilateral hemisphere of transient MCAO mice 24 hours after transient MCAO (sham = 7; WT = 7; KO = 6). Results are expressed as means ± s.e.m. (2, 3) Relative *Cd68* mRNA expression normalized to *Tbp* mRNA in cortex (sham = 6; WT = 8; KO = 8) (2) and ipsilateral caudate putamen (sham = 6; WT = 8; KO = 8) (3) 24 hours after transient MCAO. Values are expressed as fold-increases relative to values in sham-treated WT mice, which were set to “1” in each case. Results are expressed as means ± s.e.m. ns: not significant, ^*^P<0.05, ^**^P<0.01.

To investigate mechanisms underlying differences in brain damage after acute ischemia in *Angptl*2 KO and WT mice, we assessed inflammatory changes between genotypes. Compared to WT mice, *Angptl2* KO mice showed lower levels of transcripts encoding pro-inflammatory cytokines, including IL-1β and TNF-α, both of which reportedly provoke the expression of adhesion molecules and leucocyte influx in cortex and caudate putamen, at 24 hours after transient MCAO (Fig 3A and 3B).^5^ By contrast, levels of transcripts encoding IL-10, which is reported to be anti-inflammatory cytokine, [26] were comparable in ischemic brains of WT and *Angptl2* KO mice (Fig 3A and 3B). Based on this result, we assessed potential differences in macrophage infiltration or activating microglia cells in ischemic brain between genotypes, given that they can induce acute inflammation in brain ischemia.[27] To do so, we compared the number of MAC-3-positive cells in the cerebral hemisphere of WT and *Angptl2* KO mice. MAC-3 staining revealed increased macrophage infiltration into the ipsilateral caudate putamen following transient MCAO treatment of WT mice (Fig 3C and 3D1). However, there was no difference in the number of MAC-3 positive cells between two genotypes (Fig 3C and 3D1) Moreover, RT-PCR analysis revealed that transcript levels of MAC-3 and of the macrophage marker CD68 were comparable between WT and *Angptl2* KO mice in cortex and caudate putamen of transient MCAO-treated mice (Fig 3D2 and 3D3). These results suggest that attenuated brain damage after acute ischemia in *Angptl*2 KO mice is likely attributable to decreased inflammation in ischemic brain but that the outcome is not due to differences in the number of infiltrated macrophages and activated microglia.

### ANGPTL2 derived from bone marrow cells functions in progression of neuronal damage in brain ischemia

**Fig 4:**
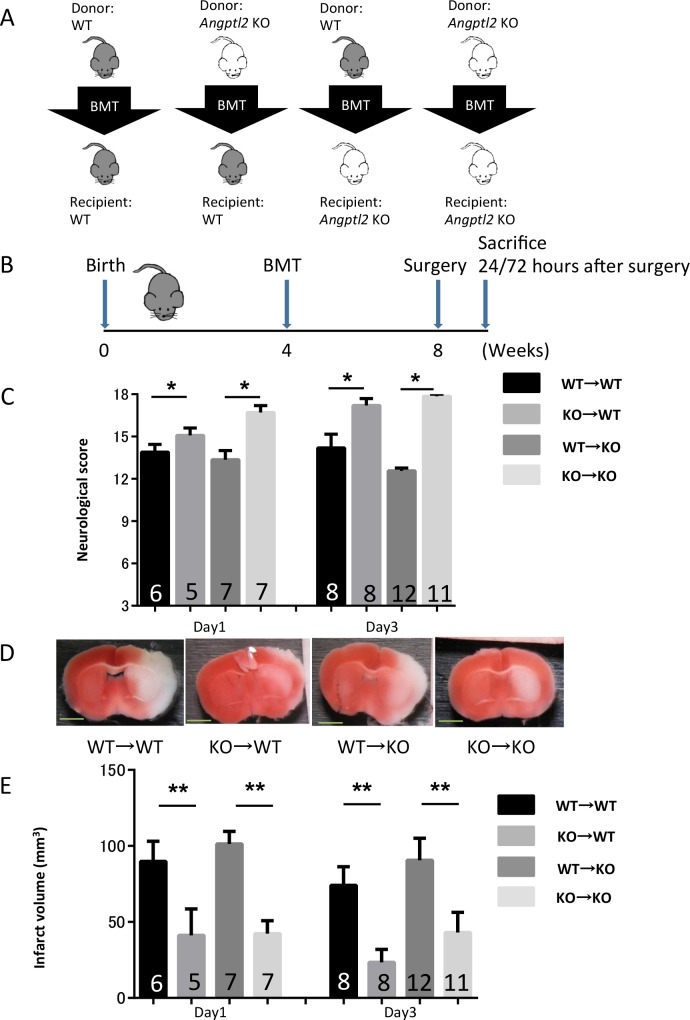
Bone marrow-derived ANGPTL2 functions in progression of neuronal damage following brain ischemia. (**A**) Schema showing relationships between donors and recipients in the bone marrow transplantation (BMT) experiment. (**B**) Schema showing the time course of BMT, surgery and sacrifice in the mouse transient MCAO model. (**C**) Graph showing neurological scores assessed by the 18-point neurological severity scale 24 and 72 hours after MCAO treatment (Day1: WT→WT = 6; KO→WT = 5; WT→KO = 7; KO→KO = 7. Day3: WT→WT = 8; KO→WT = 8; WT→KO = 12; KO→KO = 11). Results are expressed as means ± s.e.m. (**D**) Representative images showing TTC-stained brain sections 24 hours after transient MCAO in bone marrow chimeric mice. Scale bar: 2mm. (**E**) Infarct volumes as assessed by TTC-staining of brain sections 24 and 72 hours after stroke onset in bone marrow chimeric mice (Day1: WT→WT = 6; KO→WT = 5; WT→KO = 7; KO→KO = 7. Day3: WT→WT = 8; KO→WT = 8; WT→KO = 12; KO→KO = 11). Results are expressed as means ± s.e.m. ^*^P<0.05, and ^**^P<0.01.

Comparison of brain damage after acute brain ischemia between *Angptl*2 KO and WT mice suggests that ANGPTL2 modulates pro-inflammatory phenotypes and severity of brain damage after acute brain ischemia. However, major source of ANGPTL2 to promote neuronal damages in acute ischemic brain remained unclear. Therefore, we next compared contributions of ANGPTL2 from brain resident cells (choroid plexus epithelial cells, ependymal cells, or activated microglia) and bone marrow-derived cells (infiltrating macrophages) in acute brain ischemia, using bone marrow chimeric model. Particularly, we evaluated neurological deficits and infarct volume in WT or *Angptl2* KO recipient mice that had undergone transplantation with either WT or *Angptl2* KO bone marrow (Fig 4A). All recipients were irradiated at 9-Gy (a lethal dose) at 4 weeks of age, received bone marrow cells intravenously the following day, and underwent transient MCAO four weeks later (Fig 4B). We confirmed that there was no significant difference in cerebral blood flow (CBF) reduction after middle cerebral artery (MCA) occlusion among any group tested in the MCAO model (S4A Appendix). We observed little difference in infarct size between WT or *Angptl2* KO recipients that received *Angptl2* KO bone marrow (Fig 4C, 4D and 4E), indicating that brain resident cells, including ependymal cells, choroid plexus and activated microglia, are likely not major ANGPTL2 sources in the acute phase of ischemic brain. Interestingly, however, compared to WT recipients that received WT bone marrow, WT recipients that received *Angptl2* KO bone marrow showed significantly improved neurological function based on scores on an 18-point-scale neurological test and reduced infarct volume after transient MCAO (Fig 4C, 4D and 4E). In addition, compared to *Angptl2* KO recipients transplanted with WT bone marrow, *Angptl2* KO recipients transplanted with *Angptl2* KO bone marrow also showed significant attenuation of neurological deficits and decreased infarct volume (Fig 4C, 4D and 4E). These findings suggest that ANGPTL2 secreted from bone marrow-derived macrophages functions in neuronal injury in the acute phase of cerebral ischemia reperfusion injury, although macrophage infiltration in ischemic brains was not altered by *Angptl2* deletion in bone marrow (S4B Appendix).

### ANGPTL2 increases transcription of pro-inflammatory cytokines in macrophages

**Fig 5:**
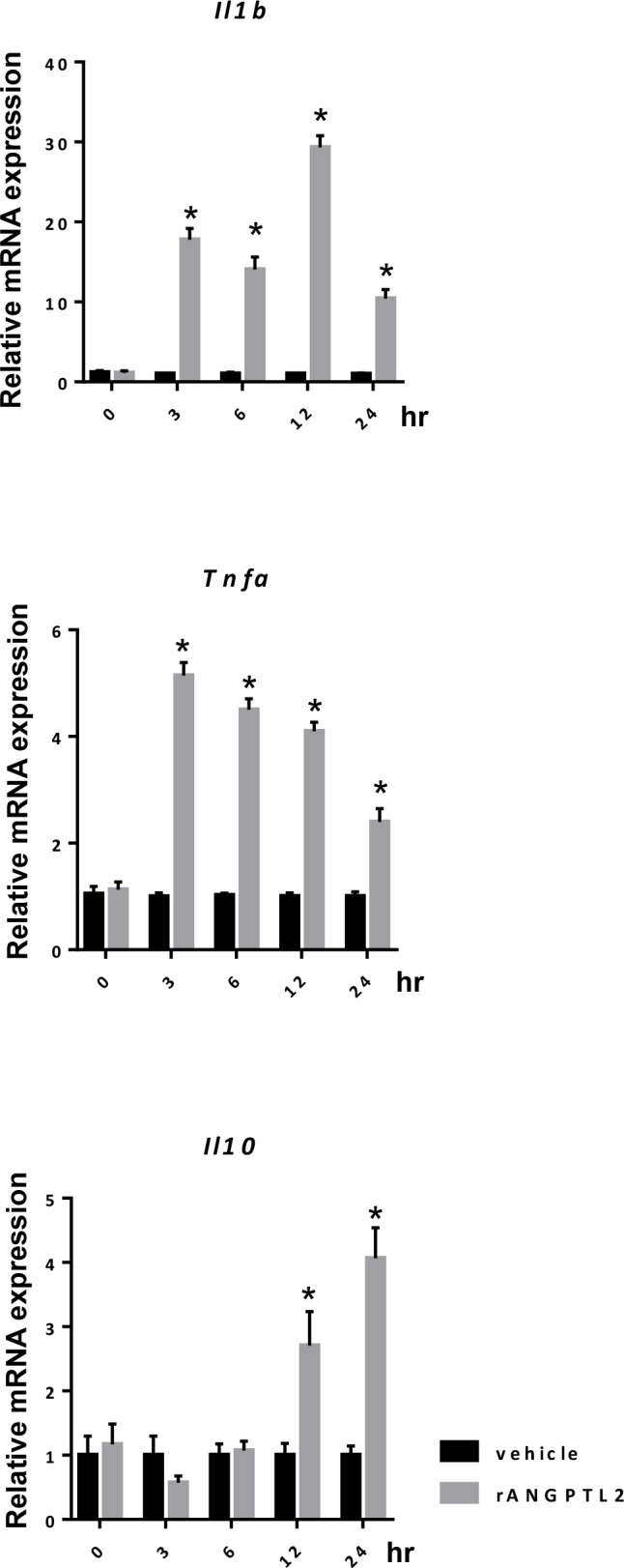
ANGPTL2 increases transcription of genes encoding pro-inflammatory cytokines in macrophages. mRNA expression levels of (left) *Il1b*, (middle) *Tnfa*, and (right) *Il10* following either vehicle or recombinant ANGPTL2 (rANGPTL2) treatment of RAW 264.7 cells (n = 5). mRNAs were harvested at 0, 3, 6, 12, 24 hours after cells were treated with 5 μg/mL rANGPTL2. Data are normalized to *Actb* levels. mRNA expression in vehicle-treated cells was set to 1 for each transcript assessed. Results are expressed as means ± s.e.m. ^*^P< 0.01.

To determine whether ANGPTL2 activates pro-inflammatory phenotypes of macrophages to increase transcription of pro-inflammatory cytokines, we performed *in vitro* analysis using the macrophage line RAW264.7. Relative to vehicle-treated controls, cells treated with recombinant ANGPTL2 (rANGPTL2) showed a rapid increase in levels of transcripts encoding pro-inflammatory cytokines including IL-1β and TNF-α (Fig 5A and 5B). Interestingly, rANGPTL2 treatment also elevated levels of IL-10 transcripts but at later time points (Fig 5C).

## Discussion

The present study reports that *Angptl2* mRNA levels increase in ischemic brain using a mouse transient MCAO model. Immunohistochemistry revealed that ANGPTL2-positive macrophages infiltrated the ischemic region but not the healthy cerebral hemisphere. In a mouse transient MCAO model employing bone marrow transplantation *Angptl2* KO and WT chimeric mice, ANGPTL2 produced by bone marrow-derived macrophages accelerated progression of neuronal damage by increasing inflammation. Furthermore, *in vitro* experiments revealed that macrophages treated with ANGPTL2 acquire pro-inflammatory phenotypes based on up-regulated levels of transcripts encoding the inflammatory cytokines IL-1β or TNF-α.

Acute inflammation reportedly perturbs neuronal function following acute ischemic stroke.[2, 28] In these cases, immune cells, including infiltrating macrophages and activated brain resident microglia, are reportedly the major intermediaries of acute inflammation.[2, 28]^,^ [29, 30] Questions have been raised about which immune cell type is primarily responsible for inflammation; however, a recent report clearly demonstrated that infiltrating macrophages rather than resident microglia were major contributors to acute inflammation in ischemic brain.[30] This finding is consistent with our finding here that bone marrow-derived infiltrating macrophages, but not resident microglia, play a crucial role in inducing acute inflammation in ischemic brain. Thus, our findings provide additional evidence for molecular mechanisms that trigger bone marrow-derived macrophages to acquire pro-inflammatory phenotypes in ischemic brain pathologies.

The integrin receptor/nuclear factor κB (NF-κB) signaling cascade is critical to activate production of pro-inflammatory cytokines by ANGPTL2.[16]^,^[17] In monocytes/macrophages, α4β2 integrin primarily and partially α5β1 integrin to some extent function to activate the NF-κB pathway.[31] Our studies lead us to conclude that ANGPTL2 increases production of pro-inflammatory cytokines including IL-1β or TNF-α in infiltrating macrophages by activating integrin/NFB signaling in acute ischemic brain. In the acute phase of ischemic stroke, pro-inflammatory cytokines damage neurons and glial cells and worsen neurological deficits.[2] IL-1β promotes immune cell migration and exacerbates disruption of the BBB and neuro-inflammation.[10] Macrophage-derived TNF-α also promotes additional inflammation by recruiting inflammatory cells,[11] thereby inducing neuronal apoptosis through TNF-related apoptosis inducing ligand.[3] Decreases in these neurotoxic effects may mediate favorable neurological outcomes observed in the ischemic brain of *Angptl2* KO mice. Therefore, lowering the cytotoxic effects of TNF-toxic and IL-1β in the CNS by ANGPTL2 suppression may serve as an attractive therapeutic option in the acute phase of ischemic stroke.

In ischemic regions, the degree of cerebral tissue inflammation is likely determined by the extent of cell damage or the number of surviving cells. Therefore, to account for these differences, we evaluated inflammatory cytokine expression not only in the “penumbra” (surrounding region) in which brain cells are reversibly damaged and potentially rescued but also in the “ischemic core,” in which they are irreversibly injured. In both regions, pro-inflammatory cytokine expression was suppressed by *Angptl2* loss. Thus, *Angptl2* loss from infiltrated macrophages attenuates acute ischemic brain damage potentially by suppressing inflammation in the acute phase of non-infectious, inflammation-associated disease. Furthermore, relevant to clinical application, attenuation of acute brain inflammation seen in the “penumbra” of *Angptl2* KO mice could be useful, as protecting the “penumbra” in stroke patients is the main purpose of therapy against acute brain infarction.[2]

**Fig 6:**
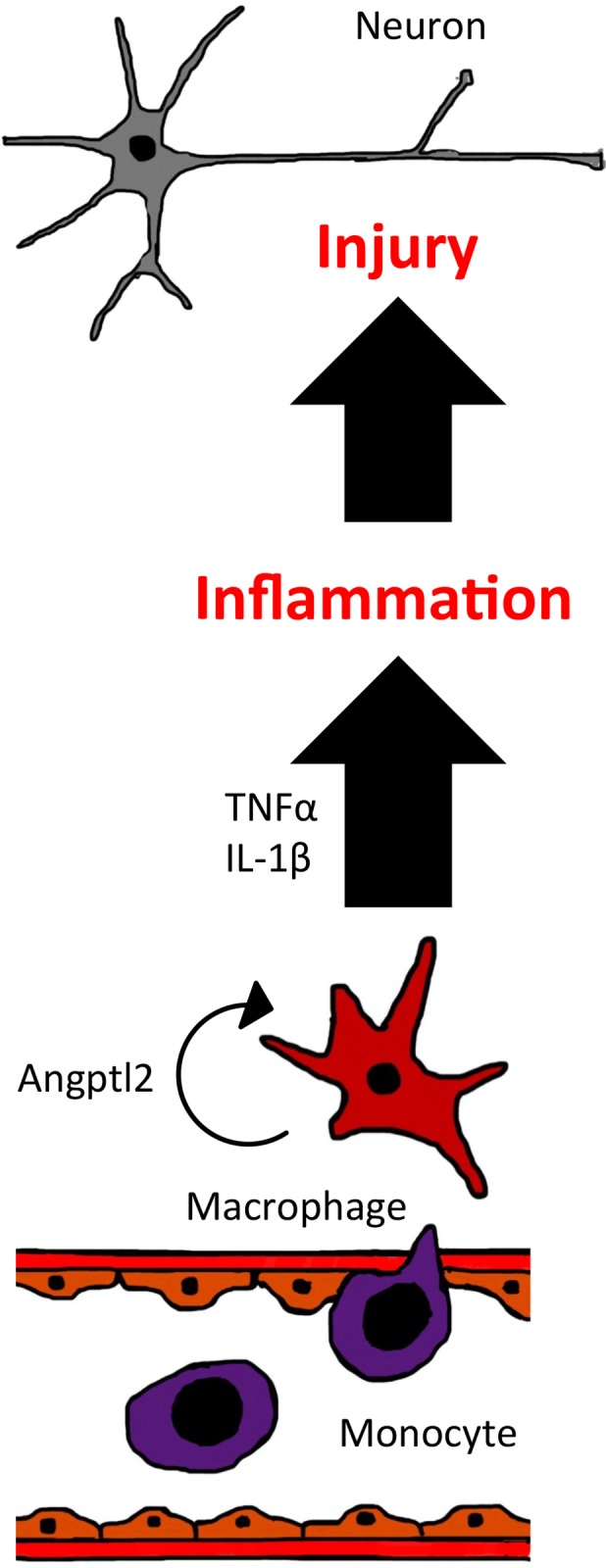
Macrophage-derived ANGPTL2 exacerbates neuronal injury in the acute phase of ischemic stroke. Schematic diagram showing ANGPTL2 function in ischemic stroke. In normal conditions, brain resident cells express little ANGPTL2. However, following ischemic stroke, bone marrow-derived macrophages infiltrate the brain interstitium and secrete abundant ANGPTL2. Subsequently, ANGPTL2 from activated macrophages induces those same macrophages to increase expression of pro-inflammatory cytokines, including TNF-α or IL-1, in an autocrine or paracrine manner. Pro-inflammatory cytokines damage neurons or supporting cells and worsen functional neurological deficits induced by brain infarction.

In both acute and chronic conditions, activated macrophages play crucial roles in the progression of several inflammatory diseases. Several previous reports indicate that ANGPTL2 is a potent activator of macrophages and enables them to acquire pro-inflammatory phenotypes in various non-infectious, chronic inflammatory diseases, such as metabolic syndrome and atherosclerotic vascular disease.[14, 16, 17] However, little evidence has been reported relevant to the role of ANGPTL2 in acute ischemic disease. A previous report indicated that ANGPTL2 derived from retinal cells or macrophages leads to leukocyte adhesion, infiltration, and expression of pro-inflammatory cytokines such as TNF-α in the acute phase of endotoxin-induced uveitis.[8] Here, we provide additional evidence that ANGPTL2 functions in acute cerebral ischemia and worsens neurological deficits, increases infarct volume, and promotes expression of neurotoxic inflammatory cytokines, including TNF-α and IL-1β (Fig 6).

Our in vitro experiments using RAW264.7 macrophages indicated that rANGPTL2 treatment promotes a gradual increase in expression of the anti-inflammatory cytokine IL-10 after a more rapid increase in levels of inflammatory cytokines, although IL-10 transcript levels were not altered by ANGPTL2 KO in the acute phase of ischemic stroke. In the progression of stroke-related brain injury, expressions of diverse cytokines in ischemic brain are changed dramatically in acute and chronic state [2]. Therefore, further investigations are needed to evaluate the effect of ANGPTL2 on brain ischemia in both acute and chronic states.

In summary, we provide the first evidence for ANGPTL2 function in the acute ischemic brain. Infiltrated macrophage-derived ANGPTL2 enhanced inflammatory responses and was associated with severe brain tissue damage in the acute phase of ischemic stroke. Our findings suggest that ANGPTL2 suppression might be a useful strategy to attenuate ischemic neuronal injury in these pathologies.

## Supporting Information

S1 Appendix This is the Supplemental data relevant to the MCAO model. (DOCX)

S2 Appendix This is the Sequences of oligonucleotide primers used for PCR. (DOCX)

S3 Appendix This is the 18-point neurological severity scale (Garcia scale). (DOCX)

S4 Appendix This is Supplemental data of MCAO in the chimeric mice. (DOCX)

S5 Appendix ANGPTL2 is expressed in choroid plexus and ependymal cells. (DOCX)

S6 Appendix Baseline vascular structure in WT and *Angptl2* KO mice. (DOCX)

S7 Appendix Parameters associated with CBF during MCAO in WT and KO mice. (DOCX)
