# Supplementary material for: Macrophage-Derived Angiopoietin-Like Protein 2 Exacerbates Brain Damage by Accelerating Acute Inflammation after Ischemia-Reperfusion
Source: PLoS One. 2016 Nov 18;11(11):e0166285. doi: 10.1371/journal.pone.0166285 (PMC5115716; doi:10.1371/journal.pone.0166285)
Supplement: S1 Appendix — (DOCX) [file pone.0166285.s001.docx]

**S1 Appendix**

**Supplemental data relevant to the mouse transient MCAO model.**

1. Exclusion criteria

Mice that met any of the following criteria were excluded:

1. Dead before evaluation
2. Surgical failure (serious blood loss or subarachnoid hemorrhage)
3. Hemorrhagic infarction
4. Complications associated with BMT
5. Sham and transient MCAO surgery of WT mice (Figure 1)

|  | Protocol | Registration | Dead | Hemorrhagic infarction or surgical failure | Analyzed |
| --- | --- | --- | --- | --- | --- |
| WT | Day 1 | 6 | 0 | 1 | 5 |

1. Transient MCAO of WT and *Angptl2* KO mice (Figure 2)

|  | Protocol | Registration | Dead | Hemorrhagic infarction or surgical failure | Analyzed |
| --- | --- | --- | --- | --- | --- |
| WT | Day 1 | 8 | 1 | 1 | 6 |
| WT | Day 3 | 10 | 0 | 2 | 8 |
| *Angptl2* KO | Day 1 | 8 | 1 | 1 | 6 |
| *Angptl2* KO | Day 3 | 10 | 1 | 2 | 7 |

1. Transient MCAO in the bone marrow chimeric mouse model (Figure 4)

Bone marrow chimeric model.
