# Supplementary material for: Macrophage-Derived Angiopoietin-Like Protein 2 Exacerbates Brain Damage by Accelerating Acute Inflammation after Ischemia-Reperfusion
Source: PLoS One. 2016 Nov 18;11(11):e0166285. doi: 10.1371/journal.pone.0166285 (PMC5115716; doi:10.1371/journal.pone.0166285)
Supplement: S2 Appendix — (DOCX) [file pone.0166285.s002.docx]

**S2 Appendix**

**Sequences of oligonucleotide primers used for PCR**

| Gene |  |  |  | Sequences |
| --- | --- | --- | --- | --- |
|  |  |  |  |  |
| *Angptl2* |  | Forward |  | GGAGGTTGGACTGTCATCCAGAG |
|  |  | Reverse |  | GCCTTGGTTCGTCAGCCAGTA |
| *Il1b* |  | Forward |  | TCCAGGATGAGGACATGAGCAC |
|  |  | Reverse |  | GAACGTCACACCAGCAGGTTA |
| *Tnfa* |  | Forward |  | AAGCCTGTAGCCCACGTCGTA |
|  |  | Reverse |  | GGCACCACTAGTTGGTTGTCTTTG |
| *Il10* |  | Forward |  | GCTCTTACTGACTGGCATGAG |
|  |  | Reverse |  | CGCAGCTCTAGGAGCATGTG |
| *Tbp* |  | Forward |  | CCCTATCACTCCTGCCACAC |
|  |  | Reverse |  | AGTTTACAGCCAAGATTCACGG |
| *Actb* |  | Forward |  | CATCCGTAAAGACCTCTATGCCAAC |
|  |  | Reverse |  | CCACCGATCCACA |
| *Cd68* |  | Forward |  | CATCAGAGCCCGAGTACAGTCTACC |
|  |  | Reverse |  | AATTCTGCGCCATGAATGTCC |
| *Pecam1* |  | Forward |  | CCGAAGCAGCACTCTTGCAG |
|  |  | Reverse |  | CTGCAACTATTAAGGTGGCGATGA |
