# Supplementary material for: Macrophage-Derived Angiopoietin-Like Protein 2 Exacerbates Brain Damage by Accelerating Acute Inflammation after Ischemia-Reperfusion
Source: PLoS One. 2016 Nov 18;11(11):e0166285. doi: 10.1371/journal.pone.0166285 (PMC5115716; doi:10.1371/journal.pone.0166285)
Supplement: S3 Appendix — (DOCX) [file pone.0166285.s003.docx]

**S3 Appendix**

**18-point neurological severity scale (Garcia scale).**

|  | score |
| --- | --- |
| **Spontaneous activity** |  |
| Moved around, explored cage | 3 |
| Moved around reluctantly, reaches at least one side of cage | 2 |
| Barely moves in cage, does not rise to any side of cage | 1 |
| Does not move at all | 0 |
|  |  |
| **Symmetry in the movement of four limbs** |  |
| All four limbs extend symmetrically | 3 |
| Limbs on contralateral side extend less than those on ipsilateral side | 2 |
| Limbs on contralateral side exhibit minimal movement | 1 |
| Forelimb on contralateral side does not move at all | 0 |
|  |  |
| **Forepaw outstretching** |  |
| Forelimbs outstretched, walking symmetrically on forepaws | 3 |
| Left side outstretched less than left, forepaw walking impaired | 2 |
| Left forelimb has limited movement | 1 |
| Left forelimb does not move at all | 0 |
|  |  |
| **Climbing** |  |
| Climbs, grips tightly with both forepaws | 3 |
| Left side impaired, does not grip as tightly and releases before right | 2 |
| Does not climb | 1 |
|  |  |
| **Body proprioception** |  |
| Reacts by turning head, equally startled by stimulus on both sides | 3 |
| Reacts slowly to stimulus on left side | 2 |
| Does not react to stimulus on left side | 1 |
|  |  |
| **Vibrissae touch** |  |
| Reacts by turning head, equally startled by stimulus on both sides | 3 |
| Reacts slowly to stimulus on left side | 2 |
| Does not react to stimulus on left side | 1 |
