# Supplementary material for: Macrophage-Derived Angiopoietin-Like Protein 2 Exacerbates Brain Damage by Accelerating Acute Inflammation after Ischemia-Reperfusion
Source: PLoS One. 2016 Nov 18;11(11):e0166285. doi: 10.1371/journal.pone.0166285 (PMC5115716; doi:10.1371/journal.pone.0166285)
Supplement: S4 Appendix — (DOCX) [file pone.0166285.s004.docx]

**S4 Appendix**

**Supplemental data associated with transient MCAO in the bone marrow chimeric model.**

1. CBF reduction rate (%) during transient MCAO in the bone marrow chimeric model.

|  | N | Before occlusion | After MCA occlusion |
| --- | --- | --- | --- |
| WT→WT | 14 | 100 | 9.535±1.145 |
| KO→WT | 13 | 100 | 9.193±0.8115 |
| WT→KO | 19 | 100 | 11.73±0.8948 |
| KO→KO | 18 | 100 | 12.60±0.9994 |

N, number of mice. Percentages indicate cerebral blood flow (CBF) reduction rate (%) before and after middle cerebral artery (MCA) occlusion (average ± standard error). Percentages of cerebral blood flow (CBF) reduction rate (%) after middle cerebral artery (MCA) occlusion (average ± standard error). Percentages before occlusion were arbitrarily set to 100%.

Number of MAC-3-positive cells in ischemic brain hemispheres in bone marrow chimeric model mice (WT→WT, n=8 ; KO→WT n=10).
