# Supplementary material for: Macrophage-Derived Angiopoietin-Like Protein 2 Exacerbates Brain Damage by Accelerating Acute Inflammation after Ischemia-Reperfusion
Source: PLoS One. 2016 Nov 18;11(11):e0166285. doi: 10.1371/journal.pone.0166285 (PMC5115716; doi:10.1371/journal.pone.0166285)
Supplement: S5 Appendix — (DOCX) [file pone.0166285.s005.docx]

**S5 Appendix**

**ANGPTL2 is abundantly expressed in choroid plexus and ependymal cells.**


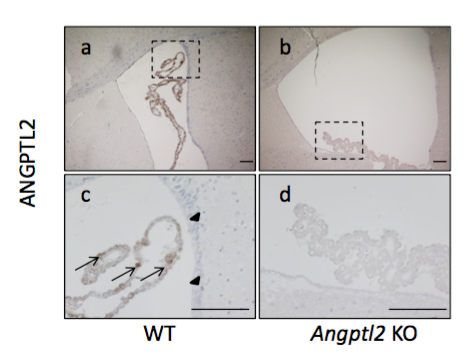


ANGPTL2 immunostaining in wild-type and *Angptl2* KO mouse brain before transient MCAO. Panels c and d are higher magnification images of squared regions in panels a and b, respectively. In c, ANGPTL2 is abundantly expressed in choroid plexus (arrows) and ependymal cells (arrowheads).
