# Supplementary material for: Macrophage-Derived Angiopoietin-Like Protein 2 Exacerbates Brain Damage by Accelerating Acute Inflammation after Ischemia-Reperfusion
Source: PLoS One. 2016 Nov 18;11(11):e0166285. doi: 10.1371/journal.pone.0166285 (PMC5115716; doi:10.1371/journal.pone.0166285)
Supplement: S6 Appendix — (DOCX) [file pone.0166285.s006.docx]

**S6 Appendix**

**Wild-type and *Angptl2* KO mice show comparable baseline vascular structure.**

**A**

**Ventral view Dorsal view**


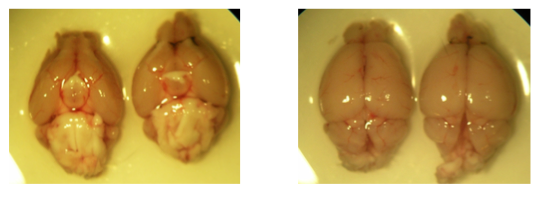


**WT *Angptl2* KO WT *Angptl2* KO**


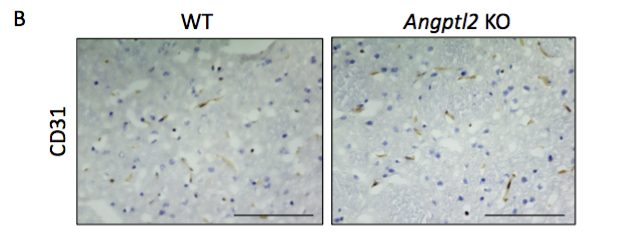


C

D

(A) Representative images showing gross structure of the cerebrovasculature in wild-type and *Angptl2* KO mice. (B) Representative images of CD31 immunostaining in striatum of wild-type and *Angptl2* KO mice. (Scale bar: 100μm) (C) Number of CD31-positive cells in ten 0.1mm^2^ areas in mouse brain sections before transient MCAO (n=5, wild-type; n=4, *Angptl2* KO). Results are expressed as means ± s.e.m. (D) Relative *Pecam1* mRNA expression levels normalized to *Tbp* mRNA in cortex and caudate putamen before transient MCAO (n=6 each). Values are expressed as fold-increases relative to values seen in WT mice, which were set to 1 in each case. Results are expressed as means ± s.e.m., ns: not significant.
