# Supplementary material for: Macrophage-Derived Angiopoietin-Like Protein 2 Exacerbates Brain Damage by Accelerating Acute Inflammation after Ischemia-Reperfusion
Source: PLoS One. 2016 Nov 18;11(11):e0166285. doi: 10.1371/journal.pone.0166285 (PMC5115716; doi:10.1371/journal.pone.0166285)
Supplement: S7 Appendix — (DOCX) [file pone.0166285.s007.docx]

**S7 Appendix**

**Parameters associated with cerebral blood flow during MCAO treatment in wild-type and *Angptl2* KO mice.**

1. CBF recovery slope

|  | N | Slope |
| --- | --- | --- |
| WT | 14 | 7.564 ± 1.380 |
| *Angptl2* KO | 13 | 6.302 ± 0.6959 |

N, number of mice. Recovery slopes were calculated using the formula: {CBF(CCAO)-CBF(MCAO)}/CBF(start)*100/CBF recovery time (min) (average ± standard error).

1. CBF recovery time

|  | N | Recovery time (min) |
| --- | --- | --- |
| WT | 14 | 8.357 ± 0.6639 |
| *Angptl2* KO | 13 | 8.462 ± 0.2683 |

N, number of mice. Recovery times (min) after reperfusion to common carotid artery occlusion level (average ± standard error).

1. CBF reduction rate (%)

|  | N | Before occlusion | After MCA occlusion |
| --- | --- | --- | --- |
| WT | 14 | 100 | 11.59±1.263 |
| *Angptl2* KO | 13 | 100 | 13.45±2.206 |

N, number of mice. Percentages of cerebral blood flow (CBF) reduction rate (%) before and after middle cerebral artery (MCA) occlusion (average ± standard error). Percentages of cerebral blood flow (CBF) reduction rate (%) after middle cerebral artery (MCA) occlusion (average ± standard error). Percentages before occlusion were arbitrarily set to 100%.
